# Supplementary material for: Longitudinal 3D Study of Anterior Tooth Wear from Adolescence to Adulthood in Modern Humans
Source: Biology (Basel). 2021 Jul 13;10(7):660. doi: 10.3390/biology10070660 (PMC8301389; doi:10.3390/biology10070660)
Supplement: Supplementary file 1 [file biology-10-00660-s001.zip › biology-1281353-supplementary.pdf]

Longitudinal 3D study of anterior tooth wear from adolescence to adulthood in modern humans

Nikolaos Gkantidis, Konstantinos Dritsas, Meret Gebistorf, Demetrios Halazonetis, Yijin Ren and Christos Katsaros

Supplementary Table S1. Spearman's correlation of the initial patient's age (T1) and the duration of the assessment period with the tooth wear amount detected for the different tooth types.

		Upper canine	Upper lateral	Upper central	Lower canine	Lower lateral	Lower central
Age at T1	Spearman's ρ	-0.07	-0.12	0.01	-0.06	-0.07	0.20
	P-value	0.553	0.339	0.962	0.649	0.605	0.143
	N	71	67	65	65	61	56
Assessment period	Spearman's ρ	0.35	0.20	0.27	0.28	0.13	-0.06
	P-value	0.003*	0.113	0.029*	0.023*	0.318	0.678
	N	71	67	65	65	61	56

*Significant correlations with $p < 0.05$

Supplementary Table S2. Differences in tooth wear (mm^3) measured for the different tooth types at the right and left sides of the mouth.

		N	Mean	SD	Mean ± SD Difference	95% Confidence Interval of the Difference		P-value*
						Lower	Upper	
Upper canine	right	64	2.77	2.51	0.42 ± 1.40	0.07	0.77	0.019
	left	64	2.35	2.29				
Upper lateral	right	54	0.78	0.97	0.06 ± 1.08	-0.24	0.35	0.695
	left	54	0.73	0.94				
Upper central	right	47	1.88	1.86	0.24 ± 1.52	-0.21	0.69	0.284
	left	47	1.64	1.45				
Lower canine	right	58	1.72	1.60	-0.10 ± 1.25	-0.42	0.23	0.562
	left	58	1.81	1.68				
Lower lateral	right	45	0.93	1.02	0.16 ± 0.59	-0.02	0.34	0.072
	left	45	0.77	0.69				
Lower central	right	38	1.48	1.36	-0.11 ± 0.90	-0.41	0.19	0.456
	left	38	1.59	1.49				

SD: Standard Deviation

*P = 0.01, Bonferroni correction applied

Supplementary Table S3. Parameter estimates indicating the effect of tested factors on tooth wear amount (dependent variable).

Parameter	B	95% Confidence Interval Lower Bound	95% Confidence Interval Upper Bound	Sig.
Intercept	-0.79	-2.51	0.92	0.364
Patient	-0.01	-0.01	0.00	0.064
Assessment period	0.19	0.08	0.30	0.001
Lower canine (ref.: upper lateral)	1.64	0.67	2.61	0.001
Lower lateral (ref.: upper lateral)	0.02	-0.97	1.00	0.972
Lower central (ref.: upper lateral)	1.28	0.32	2.25	0.009
Upper canine (ref.: upper lateral)	3.20	2.27	4.12	0.000
Upper central (ref.: upper lateral)	1.65	0.66	2.63	0.001

Female (ref.: male)	-0.93	-1.70	-0.15	0.019
Lower canine * Female (ref.: lower canine * male)	-0.83	-1.94	0.27	0.138
Lower lateral * Female (ref.: lower lateral * male)	0.20	-0.93	1.32	0.733
Lower central * Female (ref.: lower central * male)	-0.36	-1.48	0.76	0.526
Upper canine * Female (ref.: upper canine * male)	-1.61	-2.67	-0.54	0.003
Upper central * Female (ref.: upper central * male)	-0.56	-1.68	0.56	0.328

ref.: reference

Supplementary Table S4. Estimated marginal means for the factors sex and tooth type. The F tests the effect of these factors based on the linearly independent pairwise comparisons among the estimated marginal means.

	Mean	Std. Error	95% Confidence Interval		F	P - value
			Lower Bound	Upper Bound		
Tooth type						
Lower canine	2.13	0.20	1.73	2.52	20.76	0.000
Lower lateral	1.02	0.21	0.61	1.43		
Lower central	2.01	0.21	1.60	2.41		
Upper canine	3.30	0.19	2.93	3.67		
Upper lateral	0.90	0.20	0.52	1.29		
Upper central	2.27	0.21	1.86	2.68		
Sex						
F	1.21	0.08	1.05	1.37	74.57	0.000
M	2.66	0.15	2.38	2.95		

Supplementary Table S5. Spearman's correlations of tooth wear amount between the different tooth types.

		Upper canine	Upper lateral	Upper central	Lower canine	Lower lateral	Lower central
Upper canine	Spearman's ρ		0.70	0.57	0.81	0.51	0.49
	P-value		0.000*	0.000*	0.000*	0.000*	0.000*
	N		67	65	64	60	56
Upper lateral	Spearman's ρ	0.70		0.58	0.61	0.48	0.39
	P-value	0.000*		0.000*	0.000*	0.000*	0.004*
	N	67		63	61	58	54
Upper central	Spearman's ρ	0.57	0.58		0.55	0.66	0.53
	P-value	0.000*	0.000*		0.000*	0.000*	0.000*
	N	65	63		60	57	53
Lower canine	Spearman's ρ	0.81	0.61	0.55		0.62	0.48
	P-value	0.000*	0.000*	0.000*		0.000*	0.000*
	N	64	61	60		61	56
Lower lateral	Spearman's ρ	0.51	0.48	0.66	0.62		0.61
	P-value	0.000*	0.000*	0.000*	0.000*		0.000*
	N	60	58	57	61		53
Lower central	Spearman's ρ	0.49	0.39	0.53	0.48	0.61	
	P-value	0.000*	0.004*	0.000*	0.000*	0.000*	
	N	56	54	53	56	53	

*Significant correlations with $p < 0.05$
